# Whole lung lavage for pulmonary alveolar proteinosis after surgery for spontaneous pneumothorax

**Published:** 2012-09-25

**Authors:** R Stoica, A Macri, I Cordoş, C Bolca

**Affiliations:** * Department of Anesthesia and Intensive Care, “Marius Nasta” National Institute of Pneumology, Bucharest, Romania; ** 3rd Pneumology ward, “Marius Nasta” National Institute of Pneumology, Bucharest, Romania; *** Department of Thoracic Surgery, “Marius Nasta” National Institute of Pneumology, Bucharest, Romania

**Keywords:** PAS positive inclusion, secondary pneumothorax, ground-glass opacities

## Abstract

**Introduction.** Pulmonary alveolar proteinosis (PAP) is a relatively rare lung disorder, probably under diagnosed, characterized by the accumulation of lipoproteinaceosus material in the lung alveoli. The primary (acquired or idiopathic) form occurs in more than 90% of all cases. Whole lung lavage is considered the golden standard of treatment. In this report, we describe a rare case of pulmonary alveolar proteinosis with severe and incapacitating respiratory, in which whole lung lavage followed the thoracotomy for spontaneous pneumothorax.

**Case presentation.** A 34-year-old white male patient presented at the respiratory intensive care with severe respiratory failure, aggravated in the last two months, cough, night sweats and important weight loss and left spontaneous pneumothorax. The initial diagnosis of diffuse interstitial pneumopathy was revised to Pulmonary Alveolar Proteinosis after bronchoalveolar lavage. Active pleural drainage of the pneumothorax was unsuccessful and after two months, surgical suture of the lung was performed under general anaesthesia. One month later a whole left lung lavage was performed. The same procedure was also performed on the right lung. Eight months later the patient had a good exercise tolerance, normal arterial blood gas (ABG) values, and persistent ground-glass opacities in some of the pulmonary segments on CT scan.

**Conclusions. **The most severe forms of pulmonary alveolar proteinosis, in which hypoxemia and cyanosis occur, have a high mortality risk during anaesthesia and whole lung lavage. When a rare complication like spontaneous pneumothorax occurs, the suturing of the pulmonary apical blebs seems to be the only viable solution, despite the high risk of dehiscence of the sutures due to the poor pulmonary tissue integrity at the time of the whole lung lavage and during postoperative care.

## Introduction

Pulmonary alveolar proteinosis (PAP) has an estimated prevalence of 0.37 per 100 000 persons [**1**]. PAP is characterized by the accumulation of lipoproteinaceosus material in the lung alveoli. The disease is sometimes misdiagnosed because of the variable clinical course, ranging from no symptoms to severe respiratory failure and non-specific radiological aspects. The primary (acquired or idiopathic) form of the illness represents more than 90% of all cases [**2**], the other being either congenital or secondary forms (i.e. immunosuppression, hematologic diseases). Most of the patients are males with a median age at the time of diagnosis of 37.8 years and 72% were smokers at the onset of symptoms [**3**]. The golden standard of therapy in PAP is the whole-lung lavage (WLL), sequentially on both lungs. The method is laborious in a patient with high risk of life-threatening hypoxia and it is usually practiced under general anaesthesia with one-lung ventilation. The procedure is contraindicated in patients with a co-existing respiratory disease, such as respiratory infections or pneumothorax.

## Case presentation

A 34-year-old white male patient was admitted in the Respiratory ward for severe dyspnea even at rest (RR = 30 breaths/min), persistent dry cough and night sweats. His medical history revealed progressive aggravating respiratory symptoms in the last two months and important weight loss of about 15 Kg in the last 12 months. The patient was a nonsmoker with professional exposure to toxic volatiles while working in a furniture factory. Four years before he underwent treatment for cavitary pulmonary tuberculosis that he correctly completed. Physical examination revealed a white male subject with a poor general status, peripheral cyanosis, pallor, dehydration and severe protein-caloric malnutrition (Body Weight = 42 Kg and BMI = 15.1). Lung auscultation revealed attenuated breath sounds on the left side and bilateral fine end-inspiratory crackles. Oxygen saturation on air was of 72%, partially corrected at 83-85% while receiving 5 L of oxygen on facial mask. Blood gas analysis revealed hypoxemia and hypercarbia with respiratory acidosis (PaO2 =54.8mmHg, PaCO2 =69.7mmHg and pH=7.26). 

**Fig. 1:**
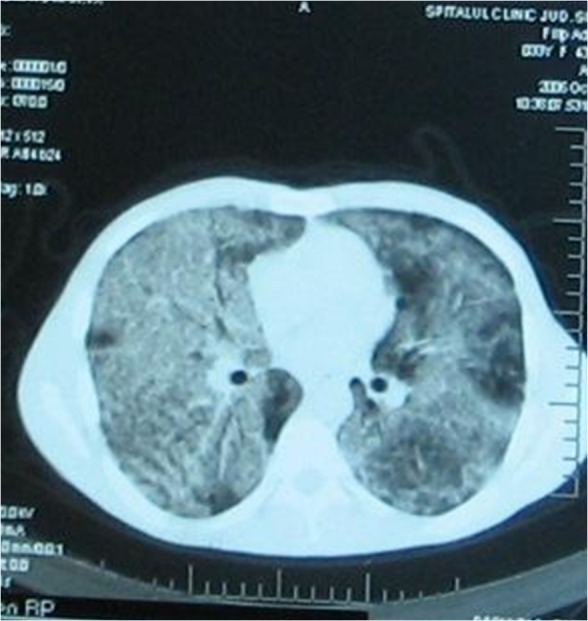
CT-scan 3 days before admission

A chest CT scan performed 72 hours days earlier during a previous admittance in another hospital described bilateral patchy airspace disease (ground-glass opacities) with ill-defined nodular or confluent pattern, interlobular septal thickening, and often perihilar predominance (**Fig. 1**).

**Fig. 2:**
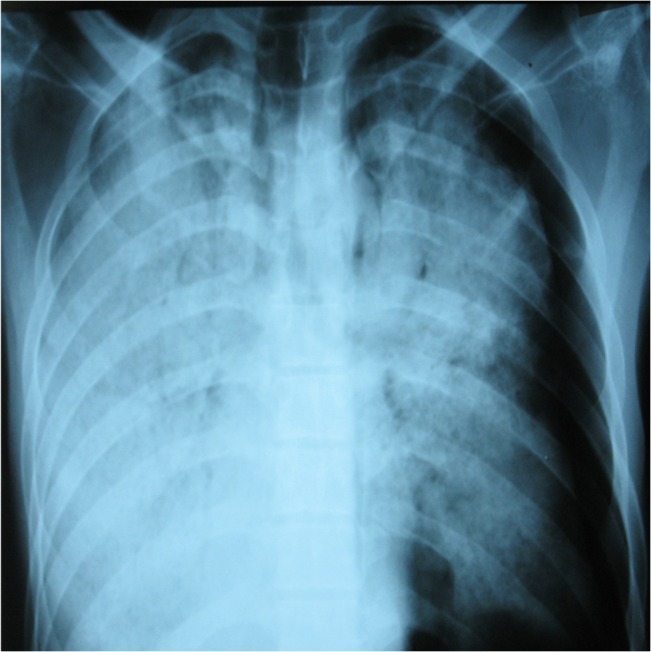
Posteroanterior chest X-ray at admission. Left pneumothorax

The chest X-ray at admission (**Fig. 2**) showed bilateral diffuse alveolar opacities with a partial left pneumothorax. Laboratory investigations revealed a high hemoglobin count (18.56g/dl), 56.4% hematocrit and hyperleukocytosis of 24.1 x 109• L-1 (mainly neutrophils).

**Fig. 3:**
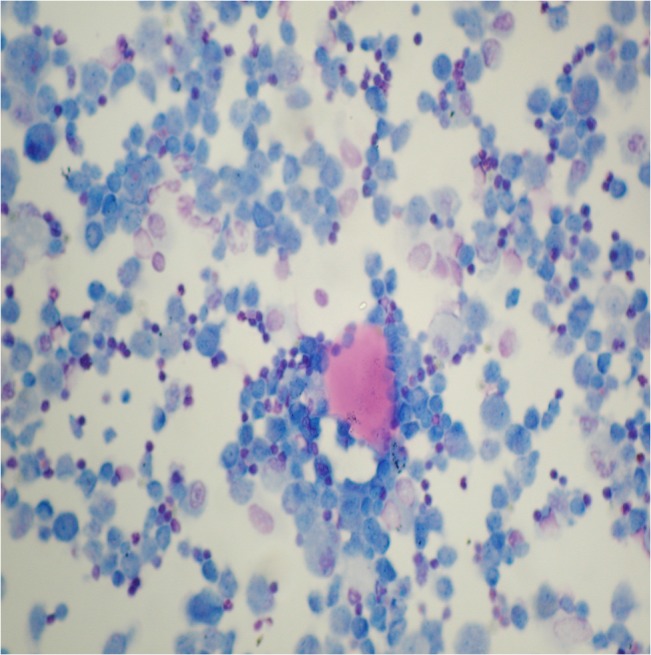
Acellular eosinophilic lipoproteinaceous material and alveolar macrophages containing PAS positive inclusion

Provisory diagnosis at this point was diffuse interstitial pneumopathy of unknown etiology, left pneumothorax, severe respiratory failure and Cachexya. The patient underwent a bronchoscopy and a bronchoalveolar lavage (BAL) with 160 ml saline, with recovery of 110 ml of milky fluid, which contained large amounts of acellular eosinophilic lipoproteinaceous material and alveolar macrophages containing PAS positive inclusions (**Fig. 3**).

At this stage, the diagnosis was defined more accurately: acquired pulmonary alveolar proteinosis (PAP), left pneumothorax with severe respiratory failure, cachexia and secondary polycythemia. The consequent clinical course of PAP was marked by a progressive deterioration of the patient’s status and the unexpected association of left lung pneumothorax that worsened the pulmonary failure and postponed the appropriate therapeutic approach in acquired PAP - the whole lung lavage (WLL). 

A sequential therapeutical approach was initiated, the first step being the surgical tube drainage of the pneumothorax. Significant pulmonary air leaks were observed following the procedure, along with an important and persistent bronchopleural fistula developing two months later. No improvements in the clinical state or lung expansion on chest X-ray images were recorded. 

**Fig. 4:**
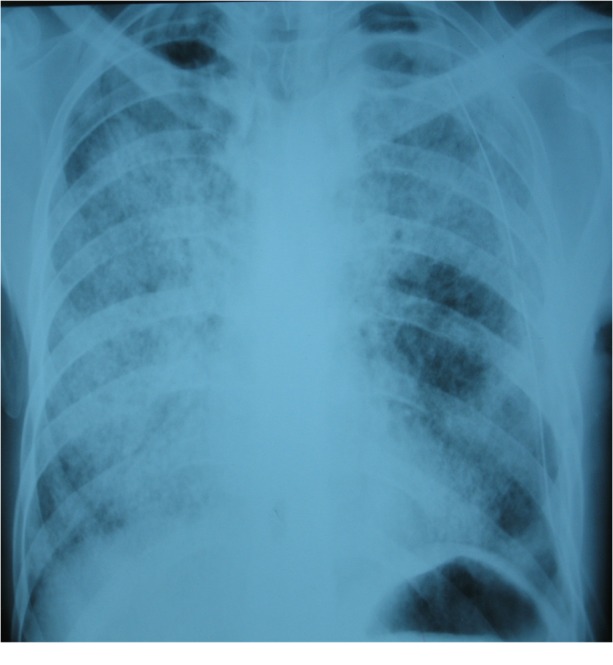
Postoperative chest X-ray after the cure of the left pneumothorax

The surgical approach for the cure of pneumothorax was delayed due to the (supposed) poor pulmonary tissue integrity, this being the main reason for dehiscence of the sutures and high mortality rate in patients with severe hypoxemia and hypercapnia after exposure to general anaesthesia. After weighing the anaesthetic and surgical risks against the risk of infection and the lack of an alternative therapy, the decision was taken to suture the bronchopleural fistula located in the apical segment of the superior left lobe under total intravenous general anesthesia (propofol + remifentanyl + atracurium), with a short period of one lung ventilation. The patient recovered well from the anaesthesia, with slightly better values of arterial blood gases one hour later. The postoperative evolution was good, no air leaks were observed and full expansion of the left lung was recorded after thoracic seal aspiration (**Fig. 4**). The thoracic drain was removed 7 days later.

Three weeks later a Whole Lung Lavage (WLL) of the left lung was performed using 15 liters of 0.9% sodium chloride solution until the return of a relatively clear fluid with no proteinaceous sedimentation. The procedure was well tolerated by the patient and recovery from the general anaesthesia took place in the OR. The values of the arterial blood gases recorded the next day showed PaO2 = 78.9 mmHg, PaCO2 = 43.6 mmHg and pH = 7.36 and SaO2 = 94.5% without supplementary oxygen. The latter CT scan also revealed an improvement in comparison with the former CT scan, showing diminished alveolar infiltrates in the left lung. 

**Fig. 5:**
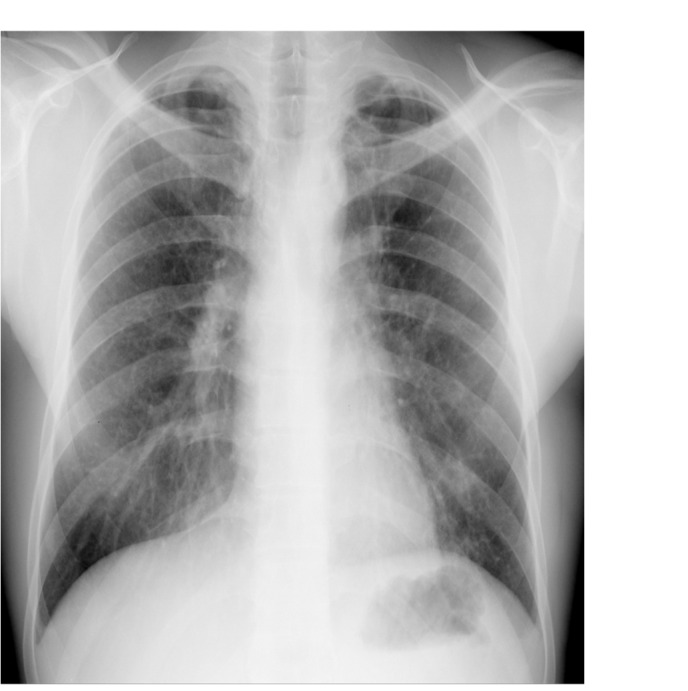
Chest X-ray after the right whole lung lavage

After two weeks, the right lung was washed with 25 liters of 0.9% sodium chloride solution. Measurements of arterial blood gases levels performed 24 hours later showed values of PaO2 = 87 mmHg, PaCO2 = 41.3 mmHg, pH = 7.422 and SaO2 = 96.8%. The chest X-ray performed 72 hours later revealed almost nonexistent pulmonary infiltrates (**Fig. 5**). 

**Fig. 6:**
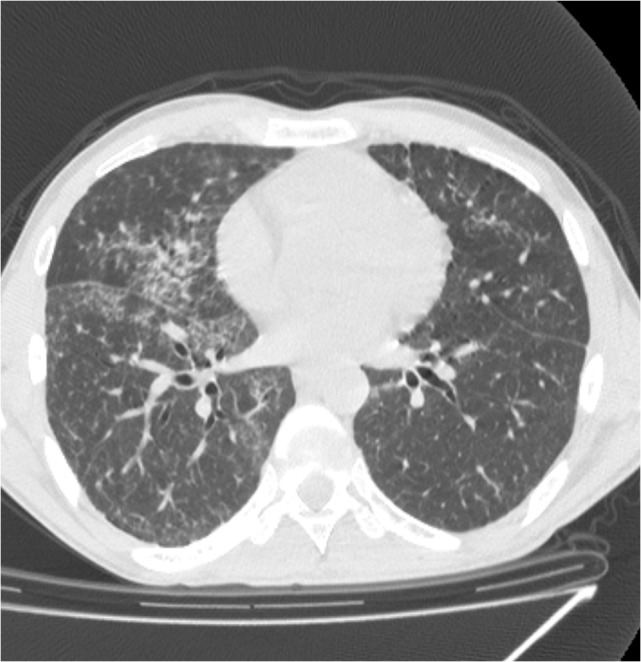
8 months follow-up CT-scan control after bilateral whole lung lavag

After eight months follow-up, the CT scan control showed persistent ground-glass opacities in some of the pulmonary segments (**Fig. 6**) while the patient had a good exercise tolerance. 

## Discussion 

For a very long time, the pathogenesis of PAP was linked with a high and constant exposure to an unknown irritant agent (i.e. silica dust) which stimulates the abnormal secretion of surfactant [**4**] or to an abnormal alveolar surfactant secretion in response to an infectious agent like Pneumocystis carinii or Cryptococcus neoformans. Later, it was proved that the pulmonary infections are in most instances a secondary event [**3**]. An important step in understanding the pathogenesis of pulmonary alveolar proteinosis was made in 1994 with the discovery of a similar pulmonary disease present in mice deficient in granulocyte-macrophage colony-stimulating factor (GM-CSF), thus revealing, as the authors mentioned, a critical role for GM-CSF in pulmonary homeostasis [**5**]. The presence of the autoantibodies against GM-GSF in the broncho-alveolar lavage fluids, inhibiting the activity of normal monocytes in patients with PAP, supports an immunologic mechanism of the disease [**6,7**]. A latex-agglutination test for GM-CSF autoantibodies with high sensitivity and specificity was developped for the diagnosis of aquired PAP [**8**]. The levels of Peroxisome proliferator-activated receptor-gamma (PPAR-gamma) are low in alveolar macrophages of patients with alveolar proteinosis and tend to normalize after treatment with GM-CSF [**9**]. The treatment of PA with subcutaneous GM-CSF is still experimental, with encouraging results in small series [**10,11**]. The diagnosis of PAP is established by BAL with PAS staining of the turbid liquid colected [**12**], and less commonly by open-lung biopsy. The management of pulmonary alveolar proteinosis depends on the progression of the illness, coexisting infections, and the degree of physiological impairment. For the last fourty years the golden standard in the treatment of PAP has been the removal of the lipoproteinaceous material by whole-lung lavage under general anaesthesia [**13**]. Patients with severe forms of PAP present with progressive clinical signs, the most common being persistent dry cough, progressive dyspnea, fatigue, weight loss, intercurrent infections etc. and "bat’s wing" or „butterfly” pulmonary opacities configuration on the chest X-ray. Cyanosis is present only in the severe forms or, like in the case presented above, when a rare complication occurs, such as spontaneous pneumothorax. This complication characterizes the unusual evolution of this case. Such a complication represents an absolute contraindication for WLL until solved. After two months of unsusccesfull chest drainage the surgical suture of the lung apical blebs was the only viable solution, despite the anaesthetic risks accompanying the severe form of pulmonary failure. The presumed preoperative poor state of the pulmonary tissue, with consequent risks of dehiscence of the sutures, was not confirmed during the surgical intervention for the cure of pneumothorax. There were no negative events during the procedure and the postoperative evolution was as expected for a normal lung. The WLL was succesfully performed 30 days after surgery, sequentially on both lungs, starting with the operated lung since the airway pressure after one-lung ventilation anesthesia is higher than the pressure used when instilling the washing liquid, which translates in a higher risk for reoccurent pneumothorax.

## Conclusions

Spontanous pneumothorax is a rare complication of PAP and because of the precarious condition of the pulmonary tissue, chest surgical drainage may be unsuccessful. Open lung surgery bears high anaesthetic and surgical risks. Full lung expansion is mandatory for the whole lung lavage, a laborious and long procedure which must be tolerated by a patient with severly impaired respiratory function. In our case the succesive surgical suture of the blebs followed by full expansion of the lung, and whole lung lavage, repeated secventially on both lungs, cleared the lungs, allowing the total recovery of the pulmonary function and, eventually, resulting in solid remission of the illness. The associated pathology and the treatment presented above have not yet been reported in literature. We conclude that surgery for pneumothorax in patients with PAP can be a solution which will later permit therapeutic whole lung lavage. 
